# Epidemiology and identification of *Eimeria* species affecting poultry in East Gojjam Zone, North West Ethiopia

**DOI:** 10.1002/vms3.1243

**Published:** 2023-08-12

**Authors:** Hailehizeb Cheru, Habtamu Tamrat, Mussie Hailemelekot, Rudi Cassini, Negus Belayneh

**Affiliations:** ^1^ Debre Tabor University Debre Tabor Amhara Ethiopia; ^2^ School of Animal Science and Veterinary Medicine Bahir Dar University Bahir Dar Ethiopia; ^3^ Department of Animal Medicine, Production and Health University of Padova Legnaro Padova Italy; ^4^ Andasa Livestock Research Center Bahir Dar Ethiopia

**Keywords:** *Eimeria* species, Ethiopia, poultry, prevalence, risk factors

## Abstract

**Background:**

Despite the expansion of modernized poultry farming in Ethiopia, the presence of high prevalence of *Eimeria* species is the bottleneck in the sector causing high morbidity and mortality rate in poultry.

**Objectives:**

The objectives of this study were to estimate the prevalence and identify *Eimeria* species and investigate the major risk factors.

**Method:**

A cross‐sectional study was conducted from November 2019 to April 2020 in East Gojjam Zone, North West Ethiopia. A total of 384 chickens were used. Both floatation and McMaster coprological techniques were employed. Univariate and multinomial logistic regression was used to calculate the odds ratio for the associated risk factors. Analysis of variance was used to analyse differences in *Eimeria* oocyst counts among the groups.

**Results:**

Overall prevalence of *Eimeria* species in poultry from the study area was 26.5%. Age (OR = 0.25, *p* = 0.001), management system (OR = 12.44, *p* = 0.001) and production system (OR = 0.37, *p* = 0.001) were found significantly (*p* < 0.05) associated with the risk of *Eimeria* species in poultry. The mean *Eimeria* oocyst count was significantly different by age and management system (*F* = 6.526, *p* = 0.002), (*F* = 5.369, *p* = 0.005), respectively. The mean *Eimeria* oocyst count was significantly greater in 6–12 weeks (*p* = 0.004) and <6 weeks of age (*p* = 0.025). A total of 6 *Eimeria* species were identified. *Eimeria tenella* (46.07%), *Eimeria necatrix* (24.5%) and *Eimeria acervulina* (8.82%) were the most common *Eimeria* species encountered.

**Conclusion:**

The prevalence of *Eimeria* species was higher in poultry in North West Ethiopia. Therefore, tailor‐made intervention is required to mitigate risk factors and reduce the prevalence of *Eimeria* species in poultry from the study area.

## Graphical abstract

Despite the expansion of modernized poultry farming in different parts of Ethiopia, the presence of high prevalence of *Eimeria* species is the bottleneck in the sector causing high morbidity and mortality rate of poultry. The aim of this study was to estimate the prevalence and identify *Eimeria* species affecting poultry and investigate the major associated risk factors. A total of 384 chickens were used. Overall prevalence of *Eimeria* species in poultry in the study area was 26.5%. *Eimeria tenella* (46.07%), *E. necatrix* (24.5%) and *E. acervulina* (8.82%) were the most common *Eimeria* species encountered.

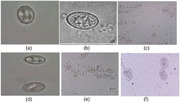

## 1 INTRODUCTION

Poultry production is one of the key livestock subsectors of Ethiopia. It plays an important roles in terms of generating employment opportunities, improving family nutrition and empowering women. It is a suitable business for low‐income households due to the small quantity of land needed and low investment costs required starting up and running the operation (Jordan, 2001). In Ethiopia, total population of chicken is estimated to be 56.38 million. Of the total population in Ethiopia, 94.31%, 3.21% and 2.49% are indigenous, hybrid and exotic breeds, respectively (Central Statistical Agency [CSA], 2017).

Despite the high number of chicken available in Ethiopia, poultry diseases are major constraints to productivity and production causing economic losses to poultry farm sector in Ethiopia. Several infectious agents like viruses, bacteria and protozoan parasites cause poultry morbidity and mortality (Alemu, 2012). *Eimeria* species are the most important protozoan parasites causing poultry coccidiosis characterized by enteritis and bloody diarrhoea (Adhikari et al., 2008).

Coccidiosis has been a major cause of poor performance and lost of productivity in poultry and other farm animals. The infectious process is rapid 4–7 days in a faecal–oral route and is characterized by parasites replication in host cells with extensive damage to the intestinal mucosa (Taylor et al., 2007). After ingestion of sporulated oocysts, sporozoites are released that enter asexual and sexual cycles of development resulting in the emergence of 1000 of new oocysts in the intestines (Taylor et al., 2007). Soon, they sporulate and become infective for chickens (Bowman, 2009). Affected birds become depressed, have ruffled feathers, the wings droop, have diarrhoea, and tend to huddle (Aarthi et al., 2010). Food and water consumption usually decreased and may become emaciated and dehydrated (Kinung et al., 2004). Laying hens will experience a reduction in the rate of egg production. Poultry coccidia are strictly host‐specific and parasitize specific parts of the intestine (Jordan et al., 2002). *Eimeria acervulina, Eimeria brunetti, Eimeria maxima, Eimeria mitis, Eimeria necatrix, Eimeria praecox and Eimeria tenella* are the most common *Eimeria* species which cause poultry coccidiosis (Taylor et al., 2007).

In Ethiopia, *E. necatrix*, *E. maxima* and *E. tenella* are endemic in all parts of the country and affect many young growing birds (Hagos et al., 2004). In the past years coccidiosis used to be the most important cause of mortality in backyard and semi‐intensive farms. An incidence of the disease was as high as 80% usually occurring in the form of outbreaks (Safari et al., 2004). Although in Ethiopia quantitative losses due to coccidiosis are not well documented, reports from other countries indicate that the disease contributes to 8.4% and 11.86% loss in profit in large‐scale farms and small‐scale farms, respectively (Lew et al., 2003).

The specific identification of *Eimeria* species and strains is important for diagnosis and control, as well as for epidemiology and biological studies of the population (Morris & Gasser, 2006). The *Eimeria* species have been identified by morphology and/or morphometry of their sporocysts and oocysts as well as their modes of development, and assessing the site and extent of the pathological lesions in the intestine of chicken (Aarthi et al., 2010). However, these methods of identifications are costly, time‐consuming, require skilled personnel and can be unreliable under the circumstances of mixed‐field infections, particularly when the overlap in biological and morphological characteristics make the unambiguous identification and differentiation of *Eimeria* species impossible (Al‐Idreesi et al., 2013; Carvalho et al., 2011). *Eimeria* oocysts can be isolated according to the method of Davies et al. (1963) and can be identified by sporulation time using potassium dichromate solution for sporulation and preservation of the parasite (Ryley et al., 1976) where molecular methods like Polymerase chain reaction are not readily available. Although quite a lot of similar studies on poultry coccidiosis have been conducted to determine the prevalence and associated risk of poultry coccidiosis in different areas of Ethiopia, it is worth noting that Ethiopia is a large country with a large number and different types of poultry farms. Therefore, most of the studies are targeting only specific areas and not the whole country but the disease is still a major problem in both backyard and semi‐intensive chicken production system in Ethiopia. There was no recent study on epidemiology and species identification of *Eimeria* species in poultry in the selected districts of East Gojjam Zone. Therefore, this study was initiated to estimate the prevalence and identify *Eimeria* species and investigate the major risk factors.

## 2 MATERIALS AND METHODS

### 2.1 Study area

The present study was conducted in three purposely selected districts of Eastern Gojjam Zone, Amhara National Regional State, Ethiopia, which is located at 10°20′ N latitude and 37°43′ E longitudes, and an altitude range of 500–4154 m.a.s.l. The mean annual rainfall of the area ranges from 900 to 1800 mm and mean minimum and maximum temperature of 7.5 and 25°C, respectively. Mixed crop‐livestock production system is a common agricultural practice in the area; backyard and semi‐intensive production is practiced in each village and household. The livestock population of the area includes 1.84 million cattle, 1.14 million sheep, 0.4 million goats, 0.09 million horses, 0.36 million donkeys, 0.014 million mules and more than 1.24 million chicken (CSA, 2017).

### 2.2 Study design

A cross‐sectional study design was conducted from November 2019 to April 2020. Both local and exotic chicken breeds which are kept under the backyard and semi‐intensive husbandry system were included in the study.

### 2.3 Sample size determination and sampling procedures

**FIGURE 1:**
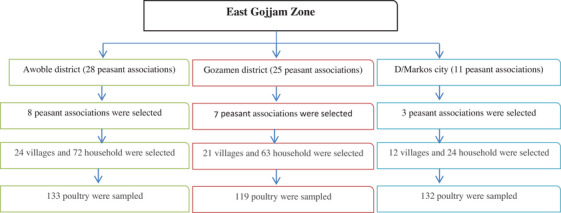
Chart showing stage of sample collection from the study areas.

The sample size used for this study was calculated according to Thrusfield (2005), assuming a 50% expected prevalence, with 95% confidence interval and 5% desired absolute precision. Accordingly, a total of 384 chickens were used for this study. The distribution of 384 samples to the districts and city was based on the number of peasant associations they have. Multistage sampling was used to select districts, peasant association, villages and household. Simple random sampling was employed to select peasant association from each districts, whereas village and household were selected purposely based on the potential availability of poultry, willingness of the owner and accessibility. Each backyard and semi‐intensive farm and chicken from each were selected by simple random sampling technique as shown in Figure 1.

### 2.4 Sample collection

About 3 g of faecal sample were collected from the upper surface of the litter immediately after the dropping of faeces using sterile disposable plastic gloves. During sampling, farm type, date of sampling, age, sex, breed, management system and production system were recorded for each chicken on a recording format. The sample was placed in a labelled clean universal bottle using 2.5% (w/v) potassium dichromate solution and transported in a cool box to Bahir Dar animal diseases diagnostic and investigation laboratory centre on the same day of collection, and preserved at refrigeration temperature until processing within 48 h of arrival.

### 2.5 Coproscopical examination

Qualitative faecal examination was conducted using flotation technique for the detection of the oocysts of *Eimeria* using concentrated sucrose solution (Sheather's sucrose solution) with specific gravity of 1.27 as described by Hendrix (1998). The slides were examined using a compound microscope (10× objectives). For identification of *Eimeria* species, positive samples were taken to the National Animal Health Diagnostic and Investigation Center, Addis Ababa, Ethiopia. Identification of *Eimeria* oocysts was based on the morphological features of the oocysts (size of oocyst and sporocysts, shape, colour and texture of oocyst wall, presence or absence of micropyle and polar cap) with the aid of taxonomic keys (Kennedy & Kralka, 1987; Sommer, 1998; Soulsby, 2005). Oocysts size was measured under ocular eye piece that is calibrated with a micrometre under a 40× objective of a microscope. Quantitative faecal examination was performed on positive samples using McMaster technique to determine the number of oocysts of *Eimeria* per gram of faeces (OPG) (Hendrix, 1998; Kaufman, 1996; Carvalho et al., 2011). The severity of the *Eimeria* infection was evaluated as mild, moderate and severe with OPG level of <1800, 1800–6000 and >6000 OPG, respectively (Carvalho et al., 2011).

### 2.6 Data management and analysis

Statistical Package for the Social Sciences (SPSS) statistical software version 20 was used to run logistic regression. Initially, the association of eight individual risk factors with an outcome variable was screened by univariate logistic regression. Those variables significantly associated with the outcome variable at *p* < 0.05 significance level in the univariate analysis were recruited for multiple logistic regression to see their independent effect. In the multinomial logistic regression analysis, a model was fitted for each outcome variable by stepwise backward elimination of insignificant variables (*p* > 0.05). ANOVA was used to analyse differences in mean oocyst count. It is not possible to perform any factor analysis for each *Eimeria* species, since the estimation of the relative percentage of each species, since only a limited number of oocyst were identified for each sample. Therefore, both presence and OPG values of the single species suffer from a high uncertainty that is going to affect the analysis if referred to single species. A statistically significant association between all variables was set at *p* < 0.05.

## 3 RESULTS

### 3.1 Association of *Eimeria* species prevalence with risk factors

A total of 384 pooled faecal samples were examined for *Eimeria* species from the 3 selected areas of East Gojjam Zone, Northwest Ethiopia. The overall prevalence of *Eimeria* species found in this study was 26.6% (102/384). In the present study, the highest prevalence of *Eimeria* species in poultry (10.2%) was recorded in Gozamen district. Besides, higher prevalence values were found in exotic breed, in younger animals, in females, and in farms characterised by a poor management and a semi‐intensive system (Table 1).

**TABLE 1:** Prevalence of *Eimeria* species in poultry in the subgroups of animals identified by the associated risk factors.

Variables categories		*N*	No of positive	Prevalence (%)
Breed	Local	136	24	6.3
	Exotic	248	78	20.3
Age	<8 weeks	148	57	14.8
	8–20 weeks	151	30	7.8
	>20 weeks	85	15	3.9
Management	Good	89	10	2.6
	Medium	182	32	8.3
	Poor	113	60	15.6
Production system	Semi‐intensive	185	70	18.2
	Backyard	199	32	8.3
Sex	Male	138	41	10.7
	Female	246	61	15.9
Districts	Gozamen	119	39	10.2
	Awobel	132	28	7.3
	D/Markos city	133	35	9.1

*Note*: *N*, number of animal sampled.

A total of six potential risk factors were tested by using multinomial logistic regression analysis. Of these, three potential risk factors were significantly (*p* < 0.05) associated with *Eimeria* species in poultry. These included animals’ age, quality of the management and production system (Table 2). Odds ratio of *Eimeria* positivity in poor management system was 12.44 times higher than good management system (Table 2).

**TABLE 2:** Potential risk factors significantly associated with the prevalence of *Eimeria* species in poultry using multinomial logistic regression analysis in the study area.

Variables	Categories	OR	95% CI	*p*‐Value
Age	<8 weeks^a^			
	8—20 weeks	0.25	0.13–0.48	
	>20 weeks	0.16	0.07–0.37	0.001
Management	Good^a^			
	Medium	3.52	1.47–8.44	0.001
	Poor	12.44	5.12–30.19	
Production system	Backyard^a^			
	Semi‐intensive	0.37	0.22–0.66	0.001

Abbreviation: OR, odd ratio.

^a^ Reference.

### 3.2 Association of *Eimeria* oocyst output with risk factors

Mean counts for each subgroup of animals and the significance of the differences are shown in Table 3. The ANOVA indicated that there was the existence of significant differences in the mean *Eimeria* oocysts counts among the age categories of poultry (*F* = 6.526, *p* = 0.002), with significantly greater counts in younger animals (age of <8 weeks), both compared to the ones with 8–20 weeks (*p* = 0.025) and to the oldest ones with >20 weeks (*p* = 0.004), as indicated by the Bonferroni pair‐wise comparison. Similarly, different management systems were significantly different in the mean count of *Eimeria* oocysts (*F* = 5.369, *p* = 0.005), with the poor management system showing higher outputs, both compared to the medium (*p* = 0.001) and to good (*p* = 0.0001) ones. However, chicken under good and medium management system did not show a significant difference (*p* > 0.05). Instead, there were no significant difference in the mean *Eimeria* oocysts count in poultry between sexes (*F* = 0.062, *p* = 0.8040), breeds (*F* = 3.108, *p* = 0.079) and production systems (*F* = 3.173, *p* = 0.076).

**TABLE 3:** Potential risk factors associated with the *Eimeria* oocyst output (OPG) using ANOVA test.

Variables	*N*	Mean	SE	*F*	*p*‐Value	Bonferroni result	*p*‐Value
**Age**							
<8 weeks	148	476.968	69.549			<8 and 8–20 weeks	0.025
8–20 weeks	151	218.358	73.314	6.526	0.002	<8 and >20 weeks	0.004
>20 weeks	85	31.415	106.441			>20 and 8–20 weeks	0.950
**Breed**							
Local	136	154.932	80.641	3.108	0.079		
Exotic	248	329.562	57.894				
**Management**							
Good	89	86.196	96.859	5.369	0.005	Good and medium	1.000
Medium	182	162.85	65.516			Good and poor	0.001
Poor	113	477.6	89.891			Poor and medium	0.001
**Sex**							
Male	138	257.700	79.830	0.062	0.804		
Female	246	233.055	62.195				
**Production system**							
Semi‐intensive	185	322.941	66.482	3.173	0.076		
Backyard	199	161.545	68.264				

### 3.3 *Eimeria* species identification

**FIGURE 2:**
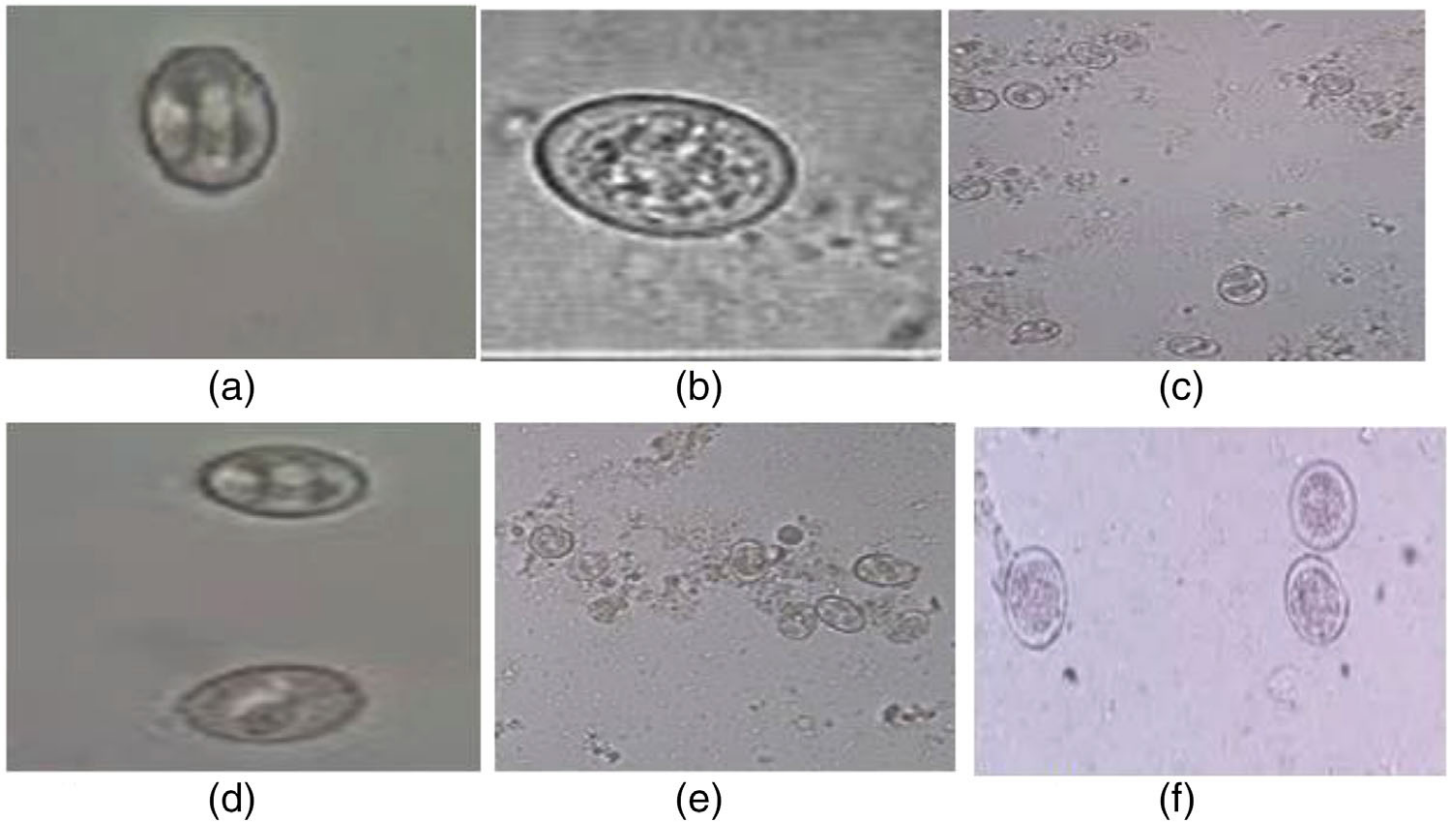
Identified *Eimeria* species; (a) *Eimeria tenella*, (b) *Eimeria mitis*, (c) *Eimeria necatrix*, (d) *Eimeria acervulina*, (e) *Eimeria maxima* and (f) *Eimeria brunetti*.

**FIGURE 3:**
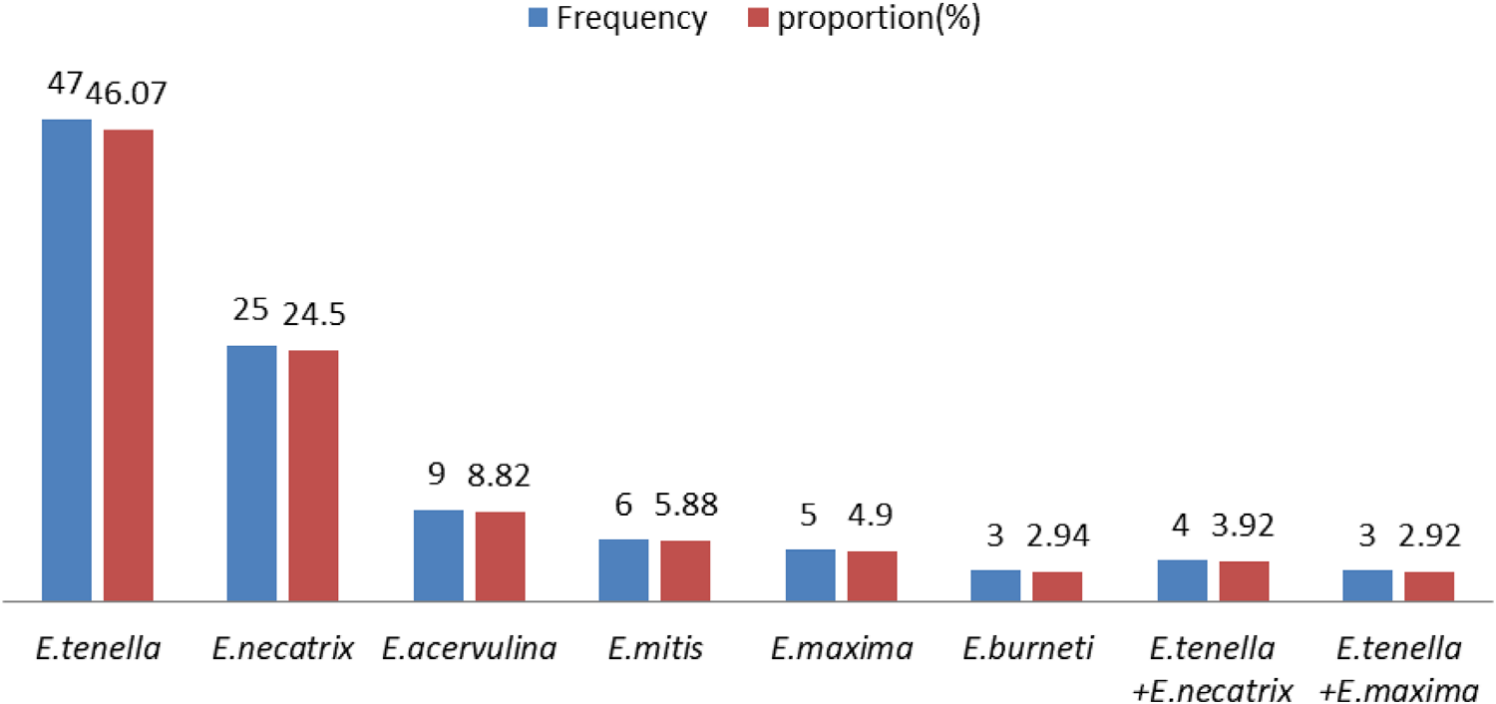
Distribution of *Eimeria* species as a single and mixed infection.

During sampling, 384 pooled faecal samples were collected according to the established protocol and 102 samples were tested positive for *Eimeria* species. For each sample the coproculture and identification of species was performed. A total of six *Eimeria* species were identified as displayed Figure 2a–f. *E. tenella* and *E. necatrix* were the more prevalent species and accounted for 46.1% and 24.5%, respectively. Other species found were *E. acervulina* (8.8%), *E. mitis* (5.9%), *E. maxima* (4.9%) and *E. brunetti* (2.9%). Mixed infections were recorded due to *E. tenella* together with *E. maxima* (2.94%), or *E. necatrix* (3.92%). The distributions of *Eimeria* species as a single and mixed infection are shown Figure 3.

## 4 DISCUSSION

In the present study, the overall prevalence of *Eimeria* species in poultry was 26.6% which is comparable to what reported in a study done in Arsi zone (22.6%) by Getachew et al. (2008) and in another study performed in central Ethiopia (25.8%) by Hagos et al. (2004). Generally, these prevalence values are halfway in the *Eimeria* species prevalence rates encountered in poultry in Ethiopia, with values ranging from about 10% to 80%. Higher prevalence values were reported by Dinka and Yacob (2012) in Debre Zeit (71.1%), by Bereket and Abdu (2015) in Kombolcha (48.5%) and by Alemargot (1987) in Addis Ababa (80.0%). At the same time, lower values were found by Fikre et al. (2005) in central Ethiopia (11.0%) and by Firmaye et al. (2015) in the same area (19.5%). The variation of these findings may be due to the differences in agro‐ecology and climatic conditions in the study areas, although most of these studies were conducted in the highland part of Ethiopia. Most probably, many confounding factors influenced the outcome of the different studies, such as different management system, seasonal variation, study design and different target animal.

Concerning risk factors, there was a significant (*p* < 0.05) association of age of chicken with the risk of *Eimeria* species infection in the present investigation. This finding is in agreement with most of the international literature, which consider the young chickens at major risk for *Eimeria* species infection (Taylor et al., 2007), and with other reports in Ethiopia. In particular, both Firmaye et al. (2015) and Hagos et al. (2004) reported higher prevalence of *Eimeria* species infection in younger than adults; and Muluken and Liuel (2017) reported a significant higher mean count of *Eimeria* oocyst in older animals. However, few other studies, both in Ethiopia (Temesgen et al., 2018) and in other countries worldwide (Julie, 1999; Muhammad, 2019), reported higher prevalence of *Eimeria* species infection in adult than in growers, either similar values. Also in this case, the presence of confounding factors, or the adoption of different age‐classes, can justify. The higher prevalence of *Eimeria* species at the age of <6 weeks is probably associated to fact that the immune system is still not fully developed at this age. Immunity to *Eimeria* species is acquired gradually and is not complete until 7 weeks of age (Allen & Fetterer, 2002). However, it might be associated also with the presence of another immunosuppressive disease, such as Gumboro diseases (Hachimi et al., 2008).

The more important risk factor for *Eimeria* species infection found in this study was the management system that resulted highly influencing the presence of the infection (animals kept with a poor management are 12.44 times at risk compared to the ones with good management) and the oocyst output (*p* < 0.01), resulting in a higher environmental contamination. This finding is in line with other reports in Ethiopia (Anteneh et al., 2019; Belaynew et al., 2016; Tekalign & Teshome, 2018) that found poor management systems at high risk of acquiring *Eimeria* species infection than good and medium management systems. This might be due to high exposure to environmental oocysts due to many managerial factors, such as lack of clean litter, poor ventilation, absence of isolation pen or defective feeders and waterers that increase the risk of oocyst ingestion. Besides, in poultry farm with poor management, the lack of vaccination for other immunosuppressive diseases like Marek's disease may reduce resistance to *Eimeria* species by interference with coccidial immunity. Consequently, coccidiosis may not respond to either preventive or curative chemotherapy in a normal way because the normal self‐limiting immunity to *Eimeria* species fails to become established.

Finally, there was difference of prevalence of *Eimeria* species in poultry also between sex, with a slightly high prevalence of coccidiosis were recorded in male than female chicken. This finding is in line with previous reports in Ethiopia, which reported higher prevalence in male than female (Anteneh et al., 2019; Kefyalew & Hailegebrael, 2018), although other studies disagree with our results (Eshetu & Nigussu, 2019) reporting a higher prevalence of *Eimeria* species in female than in male. The absence of a statistically significant difference between males and females might be due to an equal chance of exposure for the parasite infection. This indicates there is no significant biological resistance variation with the sex (Pinard‐Van Der Laan et al., 1998).

In the present study, positive samples were further investigated for *Eimeria* species identification based on morphological features of different *Eimeria* oocysts. Worldwide, more than 13 *Eimeria* species are reported in chicken. However, in the present study, six *Eimeria* species were isolated, with *E. tenella* (47%) followed by *E. necatrix* (25%) and *E. acervulina* (9%) as the most prevalent species. Except *E. mitis*, this result is in line with Solomon (2006), who reported the other five *Eimeria* species. We may consider that the six species of *Eimeria*, namely *E. acervulina, E. brunetti, E. maxima, E. mitis, E. necatrix, E. tenella*, found in this study are the most common species of *Eimeria* that infect poultry in Ethiopia.

## 5 CONCLUSION

The present study revealed that there was a prevalence of 26.6% of *Eimeria* species in poultry from the study area, which is in the middle of the prevalence values reported to date in different regions of Ethiopia. Age, management and production system of poultry were significantly associated with the prevalence of *Eimeria* species and partly with oocyst output. The average level of OPG and the widespread presence of two highly pathogenic species *E. tenella* and *E. necatrix* is particularly alarming. Based on the findings of this study, awareness creation for both the backyard and semi‐intensive poultry farm owners about all‐in and all‐out strategy and prophylactic treatment should be implemented.

## AUTHOR CONTRIBUTIONS

Conceptualization; data curation; investigation; methodology: Hailehizeb Cheru. Formal analysis; investigation; methodology; supervision; writing – original draft; writing – review and editing: Habtamu Tamrat. Supervision; writing – review and editing: Mussie Hailemelekot. Validation; writing – original draft; writing – review and editing: Rudi Cassini.

## CONFLICT OF INTEREST STATEMENT

The other authors declare no conflicts of interest.

## FUNDING INFORMATION

No funding was obtained for this study.

### ETHICS STATEMENT

The authors confirm that the ethical policies of the journal, as noted on the journal's author guidelines page, have been adhered to. No ethical approval was required, as this study required no animal experiments.

### PEER REVIEW

The peer review history for this article is available at https://www.webofscience.com/api/gateway/wos/peer‐review/10.1002/vms3.1243.

## Data Availability

The data that support the findings of this study are available from the corresponding author upon reasonable request.
